# Subclinical decelerations during developing hypotension in preterm fetal sheep after acute on chronic lipopolysaccharide exposure

**DOI:** 10.1038/srep16201

**Published:** 2015-11-05

**Authors:** Christopher A. Lear, Joanne O. Davidson, Robert Galinsky, Caroline A. Yuill, Guido Wassink, Lindsea C. Booth, Paul P. Drury, Laura Bennet, Alistair J. Gunn

**Affiliations:** 1 The Fetal Physiology and Neuroscience Group, Department of Physiology, The University of Auckland, Auckland, New Zealand

## Abstract

Subclinical (shallow) heart rate decelerations occur during neonatal sepsis, but there is limited information on their relationship with hypotension or whether they occur before birth. We examined whether subclinical decelerations, a fall in fetal heart rate (FHR) that remained above 100 bpm, were associated with hypotension in preterm fetal sheep exposed to lipopolysaccharide (LPS). Chronically-instrumented fetal sheep at 0.7 gestation received continuous low-dose LPS infusions (n = 15, 100 ng/kg over 24 h, followed by 250 ng/kg/24 h for 96 h) or saline (n = 8). Boluses of 1 μg LPS or saline were given at 48 and 72 h. FHR variability (FHRV) was calculated, and sample asymmetry was used to assess the severity and frequency of decelerations. Low-dose LPS infusion did not affect FHR. After the first LPS bolus, 7 fetuses remained normotensive, while 8 developed hypotension (a fall in mean arterial blood pressure of ≥5 mmHg). Developing hypotension was associated with subclinical decelerations, with a corresponding increase in sample asymmetry and FHRV (p < 0.05). The second LPS bolus was associated with similar but attenuated changes in FHR and blood pressure (p < 0.05). In conclusion, subclinical decelerations are not consistently seen during prenatal exposure to LPS, but may be a useful marker of developing inflammation-related hypotension before birth.

Perinatal infection is intricately linked with the adverse neurodevelopmental outcomes associated with preterm birth^1^. Infection increases the risk of preterm birth^2^ and, independently, the accompanying inflammatory response can impair neurodevelopmental outcome^1^,^3^,^4^,^5^. Currently there are limited ways to identify acute infection in its early stages. Recent clinical studies by Moorman and colleagues have shown that transient “subclinical” (i.e. mild, shallow) heart rate decelerations occur early in the course of neonatal sepsis, allowing earlier diagnosis and improved survival among very low birth weight neonates^6^,^7^. These decelerations are brief and subtle, with heart rate not falling below 100 bpm, and thus will not trigger standard heart rate alarms and may not be noticed clinically.

However, there is limited information on how this heart rate pattern is related to the development of hypotension. Additionally, it is largely unknown how infection before birth affects fetal heart rate (FHR) patterns. The risk of hypotension and death after exposure to high doses of the Gram-negative bacterial cell wall component lipopolysaccharide (LPS)^8^, is dramatically attenuated by previous low-dose LPS exposure in preterm fetal sheep^9^,^10^. This is mediated by endotoxin tolerance, whereby prior exposure to LPS reprograms the innate immune system leading to down-regulation of the pro-inflammatory responses to subsequent LPS exposures^11^.

We have recently demonstrated that the induction of tolerance by prior low-dose LPS exposure varies considerably between individual preterm fetal sheep. Despite receiving identical doses of LPS during a period of low-dose infusion, some fetuses remained normotensive after subsequent high-dose LPS boluses, whereas others developed transient hypotension, with markedly higher plasma levels of inflammatory cytokines^12^. In turn, we found that fetuses that developed hypotension showed a biphasic pattern of changes in FHR variability (FHRV), with an initial increase in FHRV after the high-dose LPS boluses, but subsequent prolonged suppression after repeated exposure to boluses. In contrast, there was no change in FHRV in fetuses that remained normotensive. These observations highlight that the speed of onset and progression of infection is likely crucial in determining the fetal cardiovascular and associated FHR responses to perinatal infection.

Given the recent clinical evidence that subclinical decelerations occur early in the course of neonatal sepsis^6^,^7^, in the present study we examined the hypothesis that brief, shallow decelerations would occur during LPS infusions, and would be related to the development of hypotension in preterm fetal sheep at 0.7 of gestation. At this age neural maturation of the sheep is broadly equivalent to 28–32 weeks of human development^13^. To test our hypothesis, we separated our cohort into those that developed hypotension (a fall in mean arterial blood pressure (MAP) by greater or equal to 5 mmHg, LPS-hypotension) and those that remained normotensive (LPS-normotension). To measure the relative frequency of subclinical decelerations, sample asymmetry was used which interrogates the relative proportion and magnitude of accelerations compared to decelerations. An increase in sample asymmetry reflects a reduction in accelerations and an increase in decelerations on FHR recordings, independent of the mean heart rate^14^.

## Methods

### Fetal surgery

All procedures were approved by the Animal Ethics Committee of the University of Auckland, and carried out in accordance with the New Zealand Animal Welfare Act, and the Code of Ethical Conduct for animals in research established by the Ministry of Primary Industries, Government of New Zealand. Singleton Romney/Suffolk fetal sheep were surgically instrumented at 98–100 days of gestation (term = 147 days) as previously reported^9^,^10^. Anaesthesia was induced by intravenous injection of Alfaxan (Alphaxalone, 3 mg/kg, Jurox, Rutherford, New South Wales, Australia), and general anaesthesia was maintained by 2–3% isoflurane in oxygen. A midline incision was made to expose the uterus, and the fetus was partially exteriorised for instrumentation. Polyvinyl catheters were placed in the left femoral artery and amniotic sac to measure MAP and amniotic pressure, respectively. Further catheters were placed in the right brachial artery and left femoral vein to allow for pre-ductal arterial blood sampling and intravenous infusions of LPS respectively. An ultrasonic flow probe (size 2R, Transonic Systems Inc., Ithaca, NY, USA) was placed around the right femoral artery to measure femoral blood flow (FBF). A pair of electrodes (Cooner Wire, Chatsworth, CA, USA) was placed subcutaneously over the right shoulder and at the level of the fifth intercostal space to measure the fetal electrocardiogram (ECG). Finally, an inflatable silicone occluder (In Vivo Metric, Healdsburg, CA, USA) was also placed around their umbilical cord for inclusion in a separate study^10^,^15^. All fetal leads were exteriorised through the maternal flank, and a maternal saphenous vein was catheterised for post-operative care and euthanasia.

Antibiotics were administered into the amniotic sac (80 mg Gentamicin, Pharmacia and Upjohn, Rydalmere, NSW, Australia) before the uterus was closed. Ewes were given 5 mL of Streptocin (procaine penicillin, 250,000 IU/mL, and dihydrostreptomycin, 250 mg/mL, Stockguard Labs, Hamilton, New Zealand) intramuscularly 30 minutes before surgery, for prophylaxis. The maternal midline skin incision was infiltrated with local analgesic (10 mL 0.5% bupivacaine plus adrenaline, AstraZeneca Ltd., Auckland, New Zealand).

### Post-operative care

After surgery, ewes were housed together in separate metabolic cages with *ad libitum* access to food and water. Rooms were temperature and humidity controlled (16 ± 1 °C, humidity 50 ± 10%) with a 12 hour light/dark cycle (light 0600 to 1800 h). Ewes were given daily intravenous antibiotics (600 mg Crystapen, Biochemie, Vienna, Austria and 80 mg Gentamicin) for four days after surgery. Fetal catheters were maintained patent with continuous infusion of heparinised saline (20 U/mL at 0.2 mL/h).

#### Data recordings

Physiological data were recorded continuously for offline analysis using custom data acquisition programs (LabView for Windows, National Instruments, Austin, TX, USA). Fetal MAP and amniotic pressure were recorded using Novatrans II, MX860 pressure transducers (Medex Inc., Hilliard, OH, USA). The fetal MAP was collected at 64 Hz, low-pass filtered at 30 Hz and corrected for maternal movement by subtraction of amniotic fluid pressure. FBF was collected using a two-channel Transonic T-206 Flowmeter (Transonic Systems Inc.) and data were 10 Hz low-pass filtered with a second order Butterworth filter. The raw fetal ECG was analogue filtered using a first-order, high-pass filter at 0.05 Hz and a low-pass, eighth-order Bessel filter at 100 Hz and digitalised at 512 Hz. From this signal continuous RR intervals were extracted for the calculation of FHR, FHRV and sample asymmetry as described below.

### Experimental Protocol

Experiments started five days after surgery, at 103–104 days of gestation. Fetuses were randomised into two experimental groups: (1) chronic saline infusion and saline boluses (saline controls, n = 8) and (2) chronic LPS infusion and LPS boluses (n = 15). This study represents an overlapping superset of our previous report^12^, including 6 and 10 previously reported animals in the saline control and LPS groups, respectively.

LPS was dissolved in saline and infused at 100 ng/kg (50 ng/mL at 83 μL/h) for the first 24 h followed by 250 ng/kg/24 h (50 ng/mL at 207.5 μL/h) for the next 96 h. Boluses were administered as 1 μg LPS dissolved in 1 mL of saline at 48, 72 and 96 h from the start of infusion. Saline controls received the same volumes of saline for both infusions and boluses. The chronic LPS infusions were chosen to cause minimal cardiovascular alterations, consistent with a subclinical infection^16^. The 1 μg LPS boluses are known to be associated with neuroinflammation but limited mortality when given after a chronic LPS infusion^9^,^15^. At 100 h after the start of infusion 4 fetuses from the saline control group and 7 fetuses from the LPS group then received complete umbilical cord occlusion for inclusion in a separate study^10^. For this reason data were only analysed until just before the third bolus, 96 h after the start of infusions.

Fetal arterial blood was collected every morning starting from 24 h before the experiment until the day of post mortem for pH, blood gases (blood gas analyser and co-oximeter 845, Ciba-Corning Diagnostics, Medfield, MA, USA), and glucose and lactate content (YSI 2300, YSI Life Sciences, Yellow Springs, OH, USA). Additional samples were taken at 2 and 6 h after the first LPS bolus and at 6 h after the remaining LPS boluses.

### Data analysis

In order to assess cardiovascular impairment following LPS administration, we compared fetuses that developed a fall in MAP of ≥5 mmHg (equivalent to a fall of ≥2 standard deviations of baseline MAP) after the first LPS bolus (LPS-hypotension, n = 8) with normotensive fetuses (LPS-normotension, n = 7), as previously described^12^. MAP and FHR were processed as 1 h averages. FHRV was assessed as the standard deviation of RR intervals (SDNN) which provides a measure of overall heart rate variability^17^. Sample asymmetry was calculated as described by Kovatchev and colleagues^14^. Briefly the median RR interval for each epoch is determined and then the raw RR intervals are separated into those greater and those less than the median. For RR intervals less than the median, the deviation of each RR interval from the median is calculated and then squared before the average of these values over the epoch is found. This final average is called R1. The same process is repeated for all RR intervals greater than the median, and the final average is called R2. Sample asymmetry is the ratio of R2/R1^14^. Both SDNN and sample asymmetry were calculated from epochs consisting of 4096 consecutive RR intervals (approximately 20 minutes of data)^6^. Each epoch was selected from the start of every 30 minutes of recording and then converted into an hourly average.

Statistical analysis was performed using SPSS (v22, SPSS Inc., Chicago, IL, USA) and SigmaPlot (v12.5, Systat Software, Washington, IL, USA). The effects of LPS infusions were evaluated by MANOVA, with time treated as a repeated measure and group as the independent factor. Holm-Sidak post-hoc tests were performed when an overall significant effect of group or a significant interaction effect between time and group was found. Statistical significance was accepted when p < 0.05. Data are presented as means ± SEM.

## Results

### Fetal biochemistry

There were no significant differences in fetal biochemistry parameters between groups during the baseline and low-dose infusion periods (Table 1). pH was significantly lower in the LPS-hypotension group at 2 hours after the first LPS bolus compared to both the LPS-normotension (p < 0.05) and the saline-control groups (p < 0.01). pCO_2_ was significantly higher in the LPS-hypotension group at 2 hours after the first bolus compared to the LPS-normotension group (p < 0.05), at 6 hours after the first bolus compared to the saline control group (p < 0.05) and at 6 hours after the second bolus compared to the LPS-normotension group (p < 0.05). Lactate was significantly higher in the LPS-hypotension group at 2 and 6 hours after the first LPS bolus compared to both the LPS-normotension (p < 0.05) and the saline control groups (p < 0.005). There were no significant changes in either pO_2_ or glucose values during the experimental period.

### Mean arterial pressure

**Figure 1:**
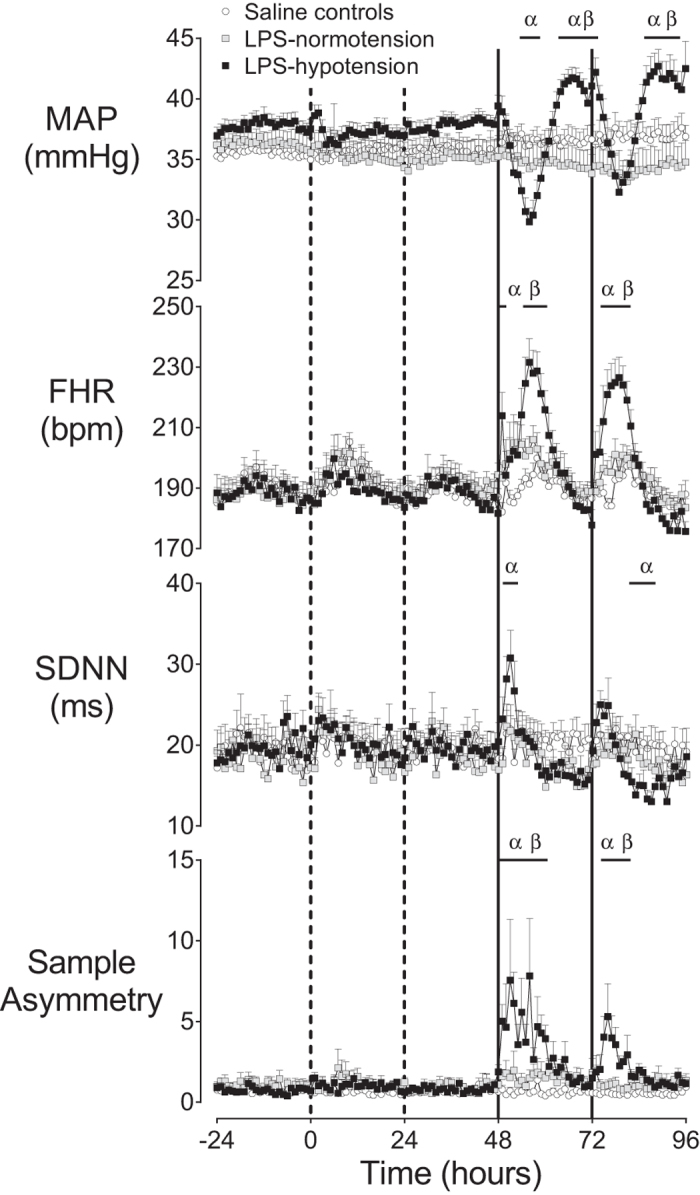
Changes in cardiovascular and heart rate patterns. Time sequence of changes in mean arterial blood pressure (MAP, mmHg), fetal heart rate (FHR, bpm), the standard deviation of RR intervals (SDNN) and sample asymmetry from 24 hours before until 96 hours after the start of infusions. Dashed vertical lines show the timing of stepwise low-dose infusions, whereas solid vertical lines show the timing of bolus administration. Data are 1 hour means ± SEM. ^α^p < 0.05, LPS-hypotension vs. saline controls; ^β^p < 0.05, LPS-hypotension vs. LPS-normotension.

There was no significant effect of low-dose infusion on MAP. In contrast, there was a significant interaction between time and group during the 48 hour period of bolus administration (p < 0.001, Fig. 1), such that MAP fell significantly in the LPS-hypotension group compared to the saline control group after the first bolus, from 54 to 58 hours (p < 0.05). MAP then progressively recovered and was significantly higher in the LPS-hypotension group compared to both the LPS-normotension and saline control groups from 64 to 73 hours (p < 0.05). A similar but attenuated pattern was observed in the LPS-hypotension group after the second bolus, but the nadir of MAP did not differ significantly between groups. MAP was significantly higher in the LPS-hypotension group compared to both the LPS-normotension and saline control groups from 86 to 94 hours (p < 0.05).

### Fetal heart rate

FHR showed a significant interaction between LPS administration and time over the 48 hour period of bolus administration (p < 0.001), such that FHR was higher in the LPS-hypotension group than both the LPS-normotension and saline control groups, as shown in Fig. 1.

### Fetal heart rate variability

There was no significant effect of low-dose LPS infusion on SDNN. However, there was a significant interaction between group and time during the 48 hour period of bolus LPS administration (p < 0.001, Fig. 1), such that SDNN increased rapidly after the first bolus in the LPS-hypotension group and was significantly higher than the saline control group from 50 to 52 hours (p < 0.05) before returning to saline control levels. After the second bolus, SDNN became significantly suppressed in the LPS-hypotension group compared to the saline control group from 82 to 87 hours (p < 0.05).

### Sample asymmetry

**Figure 2:**
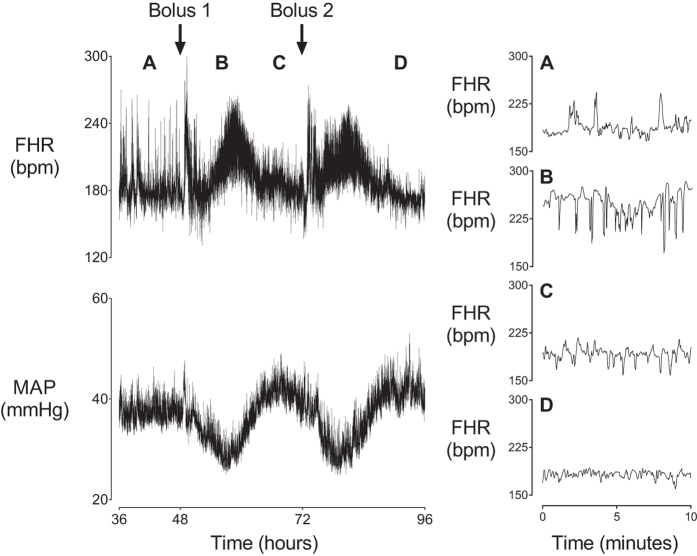
Evolving pattern of subclinical decelerations after LPS boluses. Representative heart rate traces taken from a fetus in the LPS-hypotension group from before the first bolus (**A**), approximately 4 and 18 hours after the first bolus showing the evolution of subclinical decelerations (**B**,**C**) and approximately 18 hours after the second bolus showing suppression of heart rate variability (**D**). Data in the left panels are 5 second means, data in the right panels are 1 second means.

**Figure 3:**
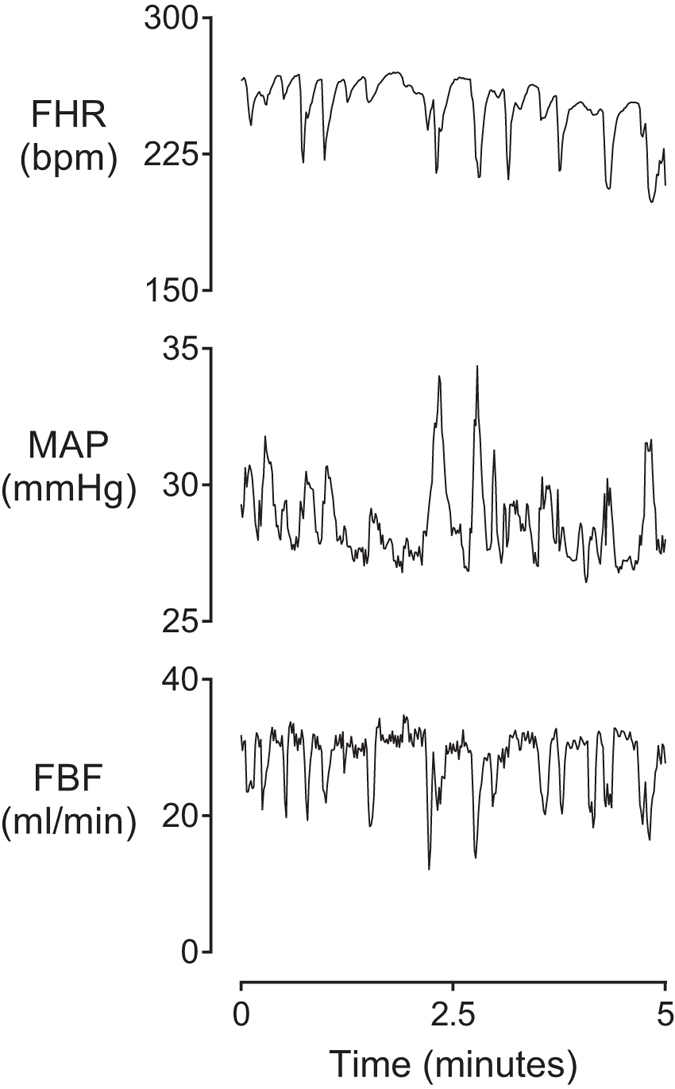
Example of immediate effects of subclinical decelerations. Example of changes in fetal heart rate (FHR, bpm), mean arterial pressure (MAP, mmHg), and femoral blood flow (FBF, mL/min) during subclinical decelerations taken from a fetus in the LPS-hypotension group approximately 10 hours after the first bolus. Data are 1 second means.

There was no significant effect of low-dose infusion on sample asymmetry. During the 48 hours of bolus administration there was a significant effect of group (p < 0.001, Fig. 1), and an interaction between group and time (p < 0.05), such that sample asymmetry increased in the LPS-hypotension group after the first bolus from 49 to 60 hours compared to both LPS-normotension and saline control (p < 0.05), before returning to saline control levels. After the second bolus, sample asymmetry increased in the LPS-hypotension group from 75 to 81 hours compared to both the LPS-normotension and saline control groups (p < 0.05) before returning to saline control levels. These increases in sample asymmetry corresponded directly with altered heart rate patterns, with frequent small amplitude decelerations and an absence of accelerations (Fig. 2). In turn, these decelerations corresponded closely in time with rapid, transient decreases in FBF and increases in MAP (Fig. 3).

### Comment

The present study demonstrates that repeated, but shallow, transient FHR decelerations after exposure to high-dose LPS in preterm fetal sheep occur only in a subset of fetuses. Strikingly, the appearance of FHR decelerations was consistently associated with the development of LPS-mediated hypotension. These transient decelerations were highly similar to the so called subclinical decelerations described in preterm neonates during developing post-natal sepsis^18^. In contrast, fetuses that remained normotensive despite high dose LPS boluses showed no heart rate changes. Consistent with our previous findings in a subset of the present study^12^, LPS-induced hypotension was associated with a biphasic pattern of FHRV. We now show that this pattern was largely mediated by the appearance of shallow, transient decelerations. These frequent decelerations corresponded closely with the initial increase in SDNN, and notably, were most frequent during the onset of the fall in fetal MAP in the LPS-hypotension group. These decelerations progressively resolved over approximately 12 hours after the first bolus with a corresponding normalization of FHRV as measured by SDNN. Interestingly, despite normalization of SDNN, the underlying FHR remained abnormal with a continued predominance of small decelerations over accelerations. Suppression of FHRV was observed after the second LPS bolus in the LPS-hypotension group, when transient decelerations had resolved.

The key finding of this study is the strong relationship between the appearance of transient, shallow decelerations and hypotension. Importantly, decelerations were seen early in the development of hypotension and persisted until after the nadir of hypotension, at a time when fetal MAP was recovering to saline-control values. This is consistent with neonatal studies that suggest that subclinical decelerations can identify infants at risk of sepsis-related mortality^7^. Nevertheless, within the LPS-hypotension group alone, there was no apparent relationship between the severity of hypotension and sample asymmetry. This may reflect that although systemic compromise and transient decelerations are initiated by similar upstream inflammatory processes, their specific mechanisms are ultimately distinct.

Previous studies have shown that inflammation-induced decelerations in mice can be abolished by atropine^19^, suggesting that subclinical decelerations in the present study may be the result of brief but intense parasympathetic activity. Moreover, there is increasing evidence that vagal activation during endotoxaemia reduces the release of inflammatory mediators, through a cholinergic anti-inflammatory system^20^,^21^. Thus, subclinical decelerations may be a side effect of activation of this potentially beneficial anti-inflammatory system. In contrast, systemic compromise and the development of hypotension during infection are most likely secondary to the release of pro-inflammatory cytokines and subsequent endothelial dysfunction^22^,^23^, likely coupled with impaired myocardial contractility^9^.

Further, we found that the majority of subclinical decelerations were associated with rapid, transient vasoconstriction of femoral arteries and a small increase in MAP. This was observed despite the progressive development of hypotension, and highlights that repeated decelerations were not the cause of hypotension. Instead, these findings suggest that subclinical decelerations are an integrative and neurally-mediated phenomenon, accompanied by a rapid reflex vasoconstriction in response to reduced cardiac output and are thus unlikely to be the result of inflammation-induced myocardial instability.

Although the second bolus of LPS was associated with only a relative fall in MAP in the LPS-hypotension group, without significant hypotension compared to saline-control levels, there was a further recurrence of transient decelerations. In contrast, the LPS-normotensive group did not show an increase in sample asymmetry (i.e. a predominance of decelerations) or background tachycardia, despite receiving the same high-dose boluses of LPS^9^,^24^. Additionally, the LPS-normotension group did not develop the mild acidosis, hypercapnia or increased lactate levels that developed in the LPS-hypotension group. We have reported that fetuses from a subset of the present LPS-normotension group showed reduced levels of inflammatory plasma cytokines^12^. Thus, these findings denote greater induction of tolerance to LPS in the LPS-normotension group from the preceding period of low-dose LPS infusion.

In our previous report, the LPS-normotension group showed a similar transient increase in FHR after LPS boluses to the LPS-hypotension group^12^. The apparent lack of increase in FHR in the expanded LPS-normotension group likely reflects minor variations in response between fetuses, with slightly more fetuses in this study showing effective attenuation of the cardiovascular responses to LPS. Enhanced development of tolerance against LPS-induced cardiovascular compromise likely also explains why decelerations were not observed in the LPS-normotension group. This is consistent with evidence from Fairchild and colleagues that bacterial and fungal infections in mice were associated with bradycardias, but that this response was rapidly desensitised by repeated exposure^19^. There is evidence that LPS exposure can attenuate the sinoatrial node’s ability to respond to parasympathetic stimulation^25^, which may have led to resistance to parasympathetic activation in the LPS-normotension group as well as progressive attenuation of decelerations in the LPS-hypotension group.

Heart rate variability represents the complex integration of oscillations of multiple different origins, including the inherent rhythms of the sinoatrial node as well as input from higher centres via the autonomic nervous system. It is through these inputs that both short and longer term oscillations, such as diurnal rhythms, are communicated to the pacemaker currents of the sinoatrial node^26^. Although heart rate and FHRV are closely related, in the present study the numerical increase in SDNN and sample asymmetry corresponded closely with marked changes in the patterns of heart rate as illustrated in Fig. 2, and the time courses of FHR and FHRV are clearly distinct. Thus it is improbable that baseline changes in FHR made a significant contribution to FHRV. Moreover, calculation of sample asymmetry involves subtraction of the individual RR intervals from the median RR interval calculated from the specific 4096 RR interval epoch that is being analysed, as detailed in the methods section. Therefore, it is inherently independent of baseline FHR. Consistent with this, there is no apparent diurnal rhythm in sample asymmetry despite the marked diurnal rhythm in FHR.

Interestingly, subclinical decelerations in the LPS-hypotension group appeared to occur in the presence of reduced background FHRV. We have previously shown that fetal body movements, which contribute to the non-neural components of FHRV^27^,^28^, were suppressed in the LPS-hypotension group early after the first LPS bolus while we also observed alterations to the FHR diurnal rhythm^12^. These findings suggest that heart rate patterns after LPS boluses reflect a loss of normal FHRV, combined with the secondary appearance of frequent decelerations which increased SDNN. Background suppression of FHRV was thus only exposed following resolution of decelerations after the second LPS bolus.

Prolonged suppression of background FHRV may be related to either a central withdrawal of autonomic activity or to impairment of the sinoatrial node. Endotoxaemia and sepsis have been associated with generalised autonomic dysfunction, which is likely related to the release of inflammatory cytokines^26^. LPS has limited ability to penetrate the blood-brain barrier^29^, however, we have previously shown that cortical electroencephalographic activity was briefly suppressed in the LPS-hypotension group, showing that systemic inflammation can have profound central effects^12^. Other *in vitro* studies have shown that LPS also has direct effects on the cells of the sinoatrial node and can interact with the hyperpolarisation-activated cyclic nucleotide-gated channels that generate the pacemaker current^30^. Such effects may impair its ability to respond to autonomic inputs.

The measurement of FHRV is complex and dependant on many factors including the particular measure and epoch length chosen. As mentioned above, SDNN did not identify the presence of abnormal FHR patterns for much of the experimental period. Given the rapid nature of the transient decelerations, other measures of short-term beat-to-beat heart rate variability may be more effective in identifying transient decelerations. This does not mean that the use of SDNN is without merit. SDNN assesses the entire distribution of RR intervals and thus provides a measure of all rhythms present in the interrogated epoch^17^. In the present study, an epoch size of 4096 RR intervals was used in order to allow for inclusion of longer term rhythms. This is likely to be appropriate for settings similar to the present study, given that inflammation appeared to be associated with a chronic loss of longer term rhythms. In contrast, although sample asymmetry is an indirect measure of subclinical decelerations, it was able to accurately detect the development of these decelerations, and proved to have superior diagnostic utility to standard time-domain measures of FHRV such as SDNN, as well as long-term variability and the root mean square of successive RR intervals, as previously reported^12^. However, sample asymmetry does not assess heart rate variability per se, and thus did not detect the suppression of FHRV that developed after the second LPS bolus. These finding illustrate the importance of using complementary measures to monitor heart rate changes.

It is important to consider that perinatal infection and neonatal sepsis are very heterogeneous in speed of onset and ultimate progression. The present study was designed to investigate fetal responses to LPS exposure using very structured doses and timings, and thus may not fully represent the dynamic nature of clinical infection. Moreover, we have only investigated the effects of Gram-negative LPS induced inflammation^31^. Nonetheless, the present findings are very similar to the heart rate changes reported during post-natal sepsis^6^. Thus this model of inflammation is consistent with some aspects of clinical infection and may be particularly suitable for studying the associated autonomic dysfunction.

The present study examines the effects of antenatal infection/inflammation, before the onset of labour. In future studies, it will be of interest to investigate how and whether inflammation affects FHR patterns during labour, when fetuses are exposed to repeated brief periods of asphyxia^32^. Clinically, antenatal suppression of FHRV is associated with adverse neonatal and neurodevelopmental outcomes^33^,^34^. Speculatively, given the persistent loss of FHRV after repeated boluses of LPS in the present study, in some cases it may be that chronic suppression of FHRV might be related to the effects of chronic infection and inflammation rather than hypoxia or intrauterine growth restriction alone. Moreover, this study illustrates that abnormal FHR patterns can often have normal overall FHRV magnitudes. This is important to consider, as moderate levels of FHRV are clinically interpreted as reassuring, with less attention paid to whether this may be associated with altered FHR patterns.

In conclusion, the present study has highlighted a strong relationship between the appearance of transient, shallow decelerations and inflammation-induced systemic compromise in preterm fetal sheep. In turn, both the shallow decelerations and hypotension could be completely attenuated by tolerance to LPS, induced by previous low-dose LPS exposure. This is an important finding as many clinical infections may have a slow progressive onset^35^, that may not affect FHR patterns. Consistent with post-natal observations in preterm infants^6^, the present study suggests that subclinical decelerations combined with suppression of background FHRV may be useful markers of the development of infection/inflammation-related hypotension before birth. These findings also highlight a potentially important limitation, that at least in this setting, these patterns did not identify fetuses exposed to high-doses of LPS that did not develop hypotension, and therefore that alternative markers are needed to identify more slowly evolving infection.

## Additional Information

**How to cite this article**: Lear, C. A. *et al.* Subclinical decelerations during developing hypotension in preterm fetal sheep after acute on chronic lipopolysaccharide exposure. *Sci. Rep.* **5**, 16201; doi: 10.1038/srep16201 (2015).

## Figures and Tables

**Table 1:** Fetal pH, blood gases and metabolites.

	Baseline	Before Bolus 1	Bolus 1 + 2 h	Bolus 1 + 6 h	Bolus 2 + 6 h
pH					
Saline control	7.37 ± 0.01	7.36 ± 0.01	7.36 ± 0.01	7.36 ± 0.01	7.36 ± 0.01
LPS-hypotension	7.37 ± 0.01	7.37 ± 0.01	7.31 ± 0.01^αβ^	7.33 ± 0.02	7.35 ± 0.01
LPS-normotension	7.38 ± 0.01	7.35 ± 0.01	7.35 ± 0.01	7.35 ± 0.01	7.37 ± 0.01
pCO_2_ (mmHg)					
Saline control	47.2 ± 0.6	48.9 ± 1.3	49.7 ± 0.9	47.6 ± 1.0	48.9 ± 1.0
LPS-hypotension	46.6 ± 1.5	47.2 ± 1.0	53.6 ± 1.7^β^	54.7 ± 1.8^α^	52.2 ± 1.0^β^
LPS-normotension	47.0 ± 1.0	48.9 ± 1.7	46.9 + 2.3	48.9 ± 2.1	47.2 ± 1.3
pO_2_ (mmHg)					
Saline control	25.7 ± 1.2	27.1 ± 1.9	26.3 ± 1.9	24.9 ± 1.9	26.7 ± 1.9
LPS-hypotension	25.7 ± 1.6	26.1 ± 1.6	24.2 ± 1.8	20.2 ± 1.2	21.7 ± 2.0
LPS-normotension	25.4 ± 1.0	25.2 ± 1.3	25.4 ± 1.7	24.2 ± 1.9	22.5 ± 1.6
Lactate (mmol/L)					
Saline control	0.8 ± 0.1	0.7 ± 0.0	0.8 ± 0.0	0.8 ± 0.0	0.8 ± 0.0
LPS-hypotension	0.9 ± 0.1	0.8 ± 0.1	1.5 ± 0.2^αβ^	2.9 ± 0.4^αβ^	1.7 ± 0.3
LPS-normotension	0.6 ± 0.1	0.8 ± 0.1	0.9 ± 0.1	1.0 ± 0.2	1.0 ± 0.4
Glucose (mmol/L)					
Saline control	1.1 ± 0.1	1.2 ± 0.1	1.2 ± 0.1	1.2 ± 0.1	1.1 ± 0.1
LPS-hypotension	1.0 ± 0.1	1.1 ± 0.1	1.1 ± 0.1	0.9 ± 0.0	1.0 ± 0.1
LPS-normotension	0.9 ± 0.2	1.0 ± 0.1	1.0 ± 0.1	1.0 ± 0.1	0.9 ± 0.1

Data are mean ± SEM. pCO_2_, partial pressure of carbon dioxide in arterial blood; pO_2_, partial pressure of oxygen in arterial blood. ^α^LPS-hypotension vs. saline control, p < 0.05. ^β^LPS-hypotension vs. LPS-normotension, p < 0.05.
